# *In vivo* evaluation of a new vena cava filter

**DOI:** 10.1590/1677-5449.006915

**Published:** 2016

**Authors:** Gilberto do Nascimento Galego, Pierre Galvagni Silveira, Júlia Jochen Broering, Eduardo da Silva Eli, Marcelo Peixer Corbellini, Amir Antônio Martins de Oliveira

**Affiliations:** 1 Universidade Federal de Santa Catarina – UFSC, Departamento de Cirurgia, Florianópolis, SC, Brazil.; 2 Universidade Federal de Santa Catarina – UFSC, Departamento de Engenharia Mecânica, Florianópolis, SC, Brazil.

**Keywords:** vena cava filter, pulmonary embolism, in vivo model, ovine model, filtro de veia cava, embolia pulmonar, modelo in vivo, modelo ovino

## Abstract

**Background:**

Pulmonary embolism is an important cause of cardiovascular death. Inferior vena cava filters have been shown to be effective for prevention of this condition.

**Objectives:**

To determine the safety, performance and efficacy of a new inferior vena cava filter in an ovine model.

**Methods:**

BKone1 filters are self-centering with over-the-wire deployment, have three filtering regions and are made from nickel-titanium alloy. Eight of these filters were implanted in 8 sheep. The sheep were divided into 4 groups of two animals (A and B) and the number of clots injected differed by group. Two clots were injected in group 2, four in group 3, eight in group 4 and zero clots in group 1. A animals underwent euthanasia soon after the procedure and B animals were observed for 30 days and then euthanized after a control cavography. All inferior vena cavas were processed for histological examination. Clots were prepared in a metal mold, sectioned and then radiopaque markers were inserted. Clot capture was analyzed by identifying the radiopaque marker on fluoroscopy.

**Results:**

No clot migration was observed during follow-up. Control cavographies showed patent inferior vena cavas. Pathological examination indicated little inflammatory tissue response. All clots were captured in the condition with 2 clots, only one clot was missed in the group injected with 4 clots and in the condition of 8 clots, they were partly captured.

**Conclusions:**

The filters were deployed safely. There was a reduction in efficacy as the number of blood clots increased.

## Resumo

**Contexto:**

Embolia pulmonar é uma importante causa de morte cardiovascular. Filtros de veia cava inferior têm se mostrado efetivos na sua prevenção.

**Objetivos:**

Determinar a segurança, o desempenho e a eficácia de um novo filtro de veia cava inferior em estudo experimental utilizando modelos ovinos.

**Métodos:**

Filtros BKone1 são autocentrantes, *over-the-wire* (OTW), compostos por três regiões de filtragem e construídos em liga de níquel-titânio. Oito filtros foram implantados em oito ovelhas. As ovelhas foram divididas em quatro grupos, de acordo com o número de êmbolos injetados, com dois animais em cada grupo (A e B). Foram injetados dois êmbolos no grupo 2, quatro no grupo 3, oito no grupo 4 e nenhum êmbolo no grupo 1. Os animais denominados A foram submetidos a eutanásia logo após o procedimento e os animais B foram observados por 30 dias, sendo submetidos a eutanásia após a realização de uma cavografia de controle. Após a eutanásia, todos os animais foram submetidos a explante do segmento de veia cava inferior contendo o filtro para análise anatomopatológica. Os êmbolos foram preparados em molde metálico e seccionados, adicionando-se marcadores radiopacos. A retenção dos êmbolos foi constatada através da identificação da marca radiopaca na seção de captura do filtro, via fluoroscopia.

**Resultados:**

Não foi observada migração do filtro após o período de 30 dias. As cavografias de controle mostraram perviedade das veias cava inferior. Os resultados dos exames anatomopatológicos indicaram pouca resposta inflamatória dos tecidos. Os êmbolos foram capturados totalmente na condição com dois êmbolos, apenas um êmbolo não foi capturado no grupo com quatro êmbolos e na condição de oito êmbolos, eles foram parcialmente capturados.

**Conclusões:**

Os filtros foram entregues com segurança. Há uma queda na eficácia de captura com o aumento da quantidade de êmbolos.

## INTRODUCTION

Pulmonary embolism (PE) is an important cause of cardiovascular death and is responsible for an estimated 120,000 to 150,000 deaths annually in the United States.^1^^,^ 2 These clots commonly arise from deep venous thrombosis and often present in patients with risk factors such as recent surgery, hospitalization, infection or malignant neoplasm.^3^ Anticoagulation remains the gold standard of care, but many patients have contraindications against this treatment or do not respond to it.^4^ In these cases surgical prophylaxis with vena cava filters^5^ is necessary.

Vena cava filters (VCF) are endovascular devices indicated for prevention of PE and death in patients for whom anticoagulation is contraindicated or ineffective.^6^ The search for a VCF with good clinical outcomes and low complication rates has prompted an increase in the number of models available on the market. An ideal device would have the following characteristics: high clot capture efficacy, non-thrombogenic properties, retrievability and absence of change to local venous flow. However, a perfect VCF has not yet been developed.^7^

**Figure 1:**
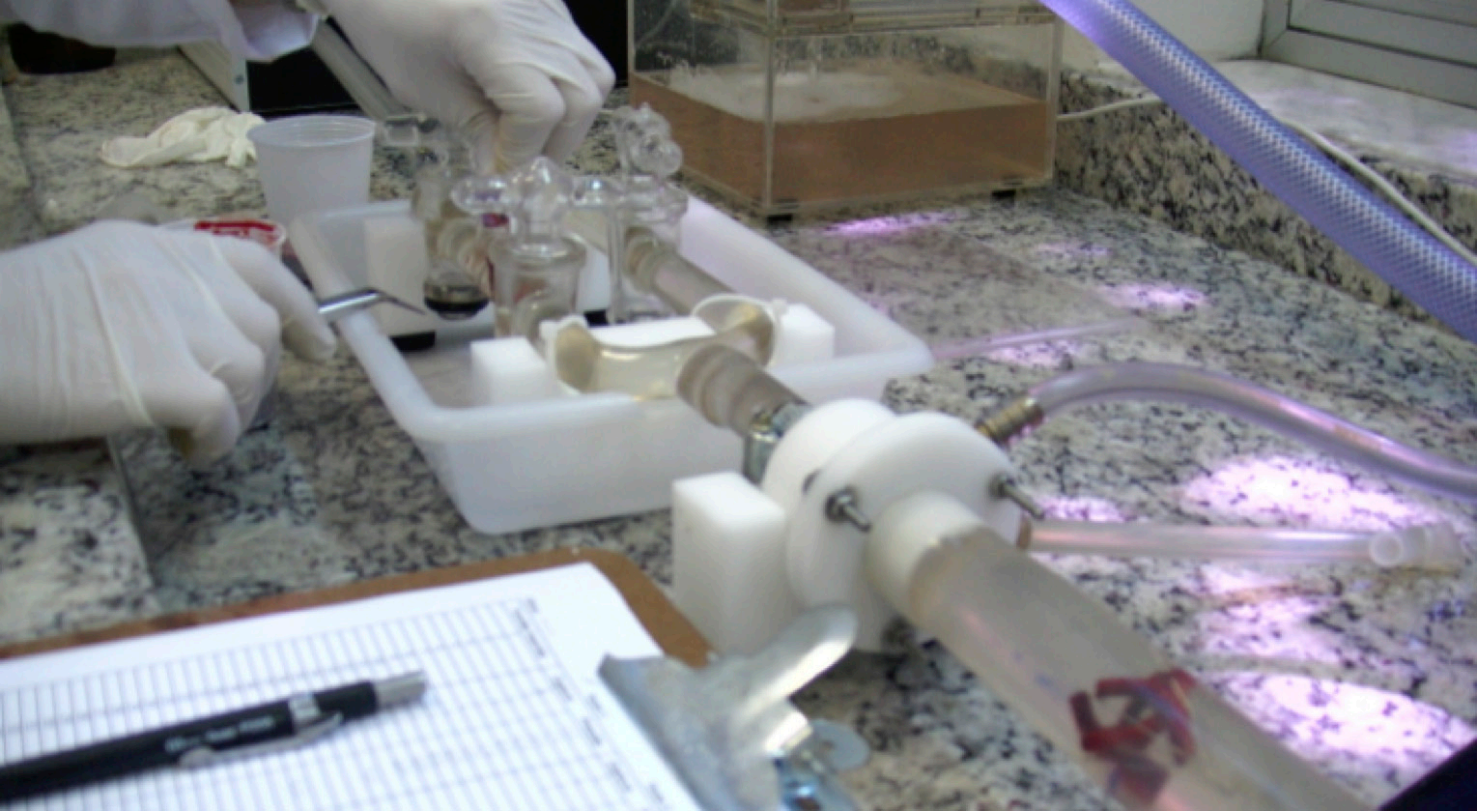
In vitro tests: experimental bench.

This study investigates a VCF prototype manufactured by Biokyra Research and Development (BKone1). The company initially designed many VCFs with different capture areas. These different designs were evaluated in tests performed on an experimental bench using a pressurized system designed to reproduced human hemodynamic conditions. The model with the best performance was selected on the basis of results for capture efficacy, change in pressure gradient with accumulation of clots and clot sizes captured (Figure 1). The filter thus selected exhibited the same capture efficacy as commercially available filters.^8^ The purpose of this study is to evaluate the safety, performance and efficacy of this filter in an ovine model.

## METHODS

### Device description

The BKone1 filter is designed for permanent placement. This filter is made of nitinol elements and is a self-centering, over-the-wire (OTW) filter with three distinct filtering regions, a proximal region, an intermediate region and a distal region.

The distal region is conical in shape and is made up of 0.25 mm wire rods with limited penetration. Its primary function is to capture clots (filtering), and its secondary function is to provide anchorage to prevent filter migration. When the filter is in the maximum diameter of 35 mm, it is 45 mm height.

The intermediate region has an inverted cone shape and is connected to the proximal region, which is also conical in shape. The main function of these regions is to centralize the filter, but they are also designed to retain clots that have not been captured by the distal region.

**Figure 2:**
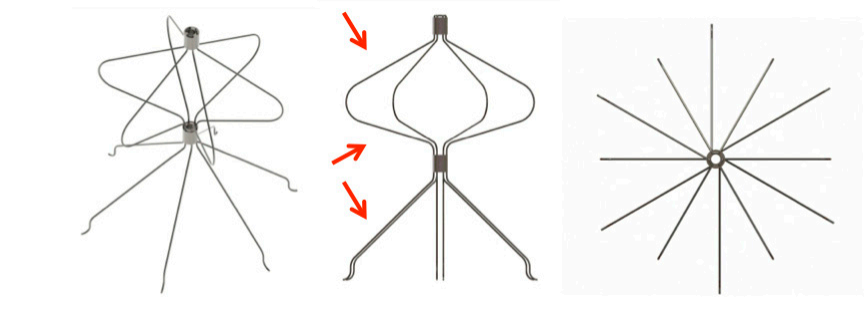
BKone1 filter in the upper oblique view, from the side and from below, respectively. Central picture: arrows show the three capture regions.

The rods are joined mechanically by deformation of a stainless steel sheet 3 mm in length, creating an interference fit at the junction between the distal and the intermediate region, and another similar 3 mm piece joining the proximal ends of the rods, also by interference (Figure 2).

The filter can be delivered via either femoral or jugular access. Femoral access was chosen for these tests. The device is delivered through a catheter introducer sheath (12Fr internal diameter) with an introducer catheter (600 mm), containing an hemostatic valve dilator catheter (670 mm), a pusher catheter (700mm) and a guide wire (0.035” × 1500mm).

**Figure 3:**
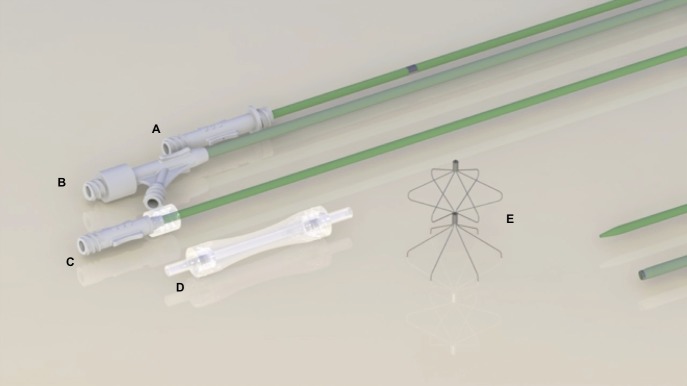
VCF and delivery system shown from top to bottom: (A) pusher catheter; (B) catheter introducer sheath; (C) dilator catheter; (D) cartridge containing VCF; (E) VCF.

BKone1 comes inside a transparent polycarbonate cartridge. There is a tube within the guide wire path that directs the passage of the guide wire. There is also a security lock at each end of the cartridge to hold the guide wire tube in the correct position. The position, femoral or jugular, is indicated on the cartridge (Figure 3).

### Animal groups

This study was approved by the Ethics Committee at the Albert Einstein medical school. Eight Santa Ines sheep, each weighing between 42.5 and 75 kg and with vena cava diameters ranging from 1.3 to 4.0 cm, were distributed randomly into 4 groups (1, 2, 3 and 4), two animals per group (A and B), and the number of clots injected after VCF placement differed by group. Group 1 was a control group and so zero clots were injected in the animals in this group. Two clots were injected in group 2, four in group 3 and eight in group 4. Animals designated A were euthanized shortly after the procedure and animals designated B were observed for at least 30 days before euthanasia.

### Clot preparation

**Figure 4:**
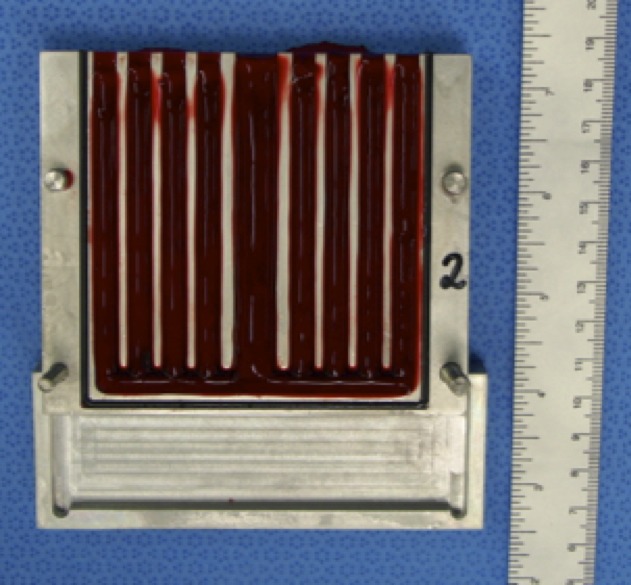
Mold built specifically for this study, filled with blood.

Around 10-20 ml of blood was collected by intravenous puncture of sheep from groups 2, 3 and 4 on the day before VCF placement. The blood collected was then used to fill a metal mold built specifically for this study and sterilized in advance. The mold has 2 parts, each 9 cm long, and was designed with furrows that create a space 5 mm in diameter when the two parts are joined together. After 24 hours, the two parts of the mold were separated (Figure 4).

**Figure 5:**
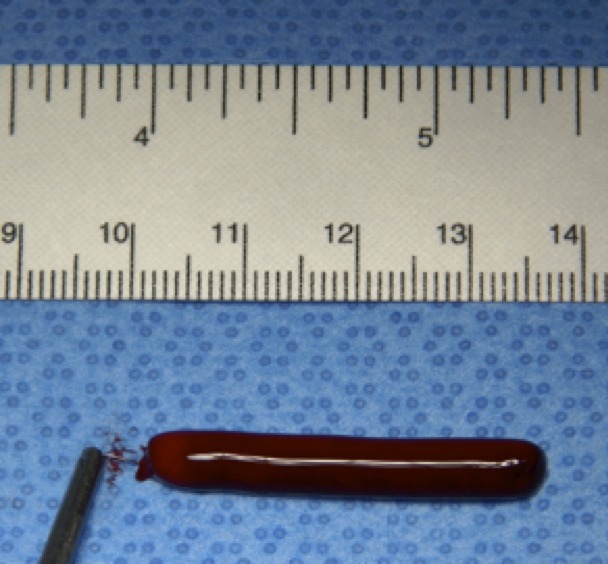
Insertion of the platinum wire into one extremity of a clot already cut to size.

Clots were removed from the mold and cut to a standard length of 35 mm. Two pieces of platinum wire, each 5 mm long and with a diameter of 0.2 mm, were inserted into the extremities of each clot (Figure 5). The clots were then drawn up into a 14 Fr sheath one by one to serve as a source of emboli.

### Filter placement

All the procedures were performed in a Hemodynamic Laboratory in the Training and Research Center at the Instituto Israelita de Ensino e Pesquisa Albert Einstein, São Paulo, SP, Brazil. The anesthetic procedure was initiated by a veterinarian using intramuscular ketamine (10 mg/kg dose) and midazolam (0.25 mg/kg). One of the animal's ear veins was punctured and propofol was administered (7 mg/kg) to aid positioning into a supine position, and then tracheal intubation was performed. A capnograph was connected between the endotracheal tube and the ventilation circuit. Anesthesia was maintained using isoflurane in concentrations of 2 to 4% in a closed system with oxygen and compressed air flow set to 1.5 to 2 L / min.

An orogastric tube was inserted to facilitate decompression of the rumen and a pulse oximetry probe was placed on the tongue, ear, lip or nipple for monitoring. Temperature, heart rate and respiratory rate were recorded throughout anesthesia.

After induction of anesthesia, the right common femoral vein was identified and punctured with the aid of Doppler ultrasonography. Using the Seldinger technique, a 0.035 guide wire was progressed up to the VCI and then the (12Fr) introducer system (also developed by Biokyra Research and Development) was inserted. At this point, the veterinarian injected the animal with 1ml (50mg) of sodium heparin to prevent coagulation. A cavography was performed with injection of 20 ml of contrast (Telebrix 30 Meglunina) for anatomical study and to locate the renal veins.

The distal extremity of the sheath was positioned below the renal vein and the dilator was removed. Next, the filter was docked at the distal extremity of the sheath and, with the aid of a pusher catheter, was placed and released into the infrarenal segment by a pull-back mechanism. After deployment, a second cavography was performed to confirm that the VCF was correctly placed and the IVC was patent.

For animals in groups 2, 3 and 4, once the VCF had been delivered, the 12 Fr system was removed and an 18 Fr introducer was inserted and positioned in line with the iliac bone and the guide wire was removed. The 14Fr sheath containing the clots was then introduced into this 18Fr introducer.

**Figure 6:**
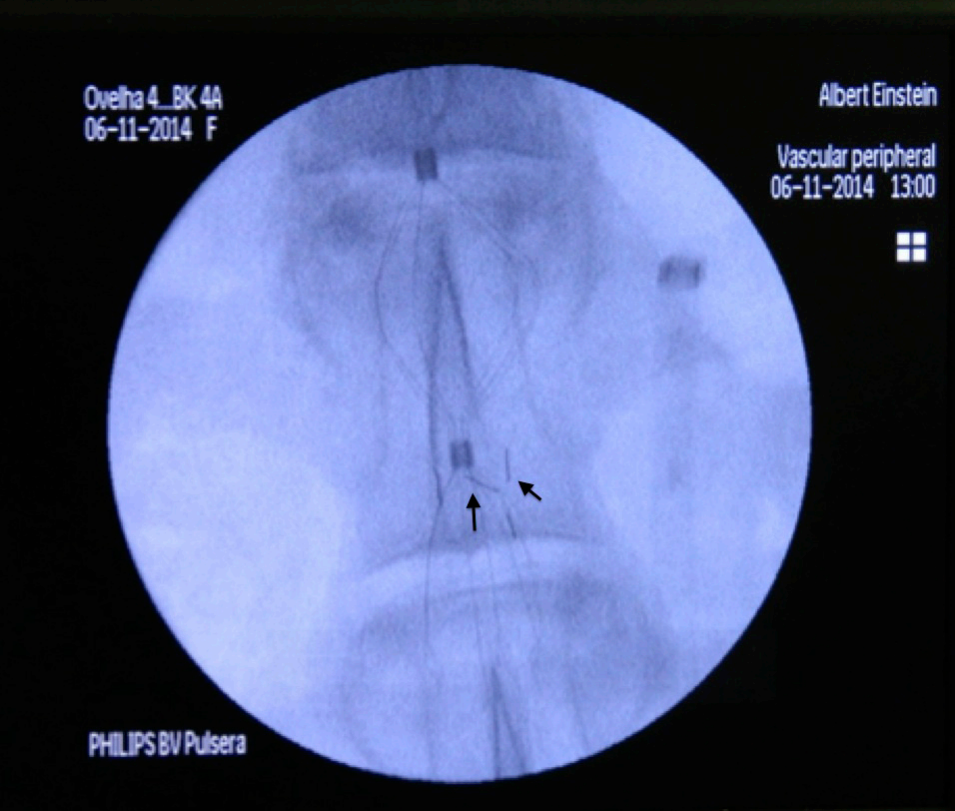
Time fluoroscopy image showing clots captured by the VCF (which can be located by their respective radiopaque markers, as indicated by the arrows).

Clots were injected into the iliac vein with 20 ml of 0.9% saline solution, slowly and progressively, using direct fluoroscopy visualization to monitor their trajectory. Fluoroscopies were conducted of the thoracic region in order to locate non-captured clots (Figure 6).

For B animals, 1ml of protamine hydrochloride diluted in 20 ml of 0.9% saline solution was injected over a period of 1 minute in order to neutralize the heparin. These animals were brought out of anesthesia and were observed for a period of at least 30 days.

Group A animals were subjected to induction of deep anesthesia and painless death was effected by anesthetic overdose and hypovolemia. Hypovolemia was achieved by puncturing the femoral artery and connecting it to a vacuum system. After euthanasia, a laparotomy was performed and an IVC segment of approximately 7cm containing the VCF was removed. This specimen was stored in 10% formaldehyde and sent for histopathological analysis.

Group B animals were observed to evaluate their post-operative condition and to detect possible complications resulting from the procedure. After the follow-up period, these animals were transported to the hemodynamic laboratory and subjected to the same anesthetic protocol as in the first procedure. A cavography was performed to evaluate IVC patency and filter position and compared to the cavography from day 1.

**Figure 7:**
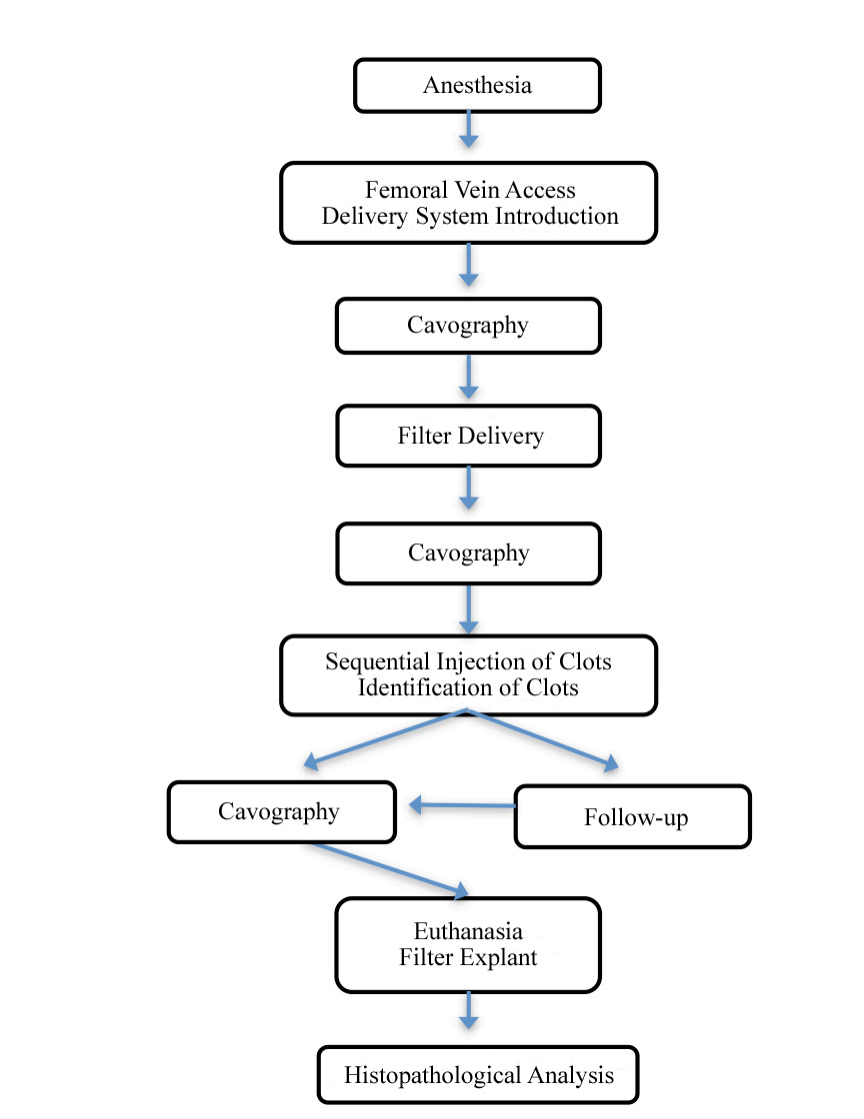
In vivo test summary: Group A animals were subjected to euthanasia after cavography and VCF delivery; Group B animals were followed for at least 30 days, subjected to a second cavography and subsequently euthanized. The VCI segment containing the VCF was sent for histopathological analysis after explantation.

After this procedure, the animals were euthanized and the IVC segment containing the filter was removed, as had previously been performed with A animals. This specimen was stored in 10% formaldehyde and sent for histopathological analysis. Figure 7 summarizes the methods.

## RESULTS

Delivery of the VCFs using the dedicated delivery system, developed by Biokyra Company, proved to be feasible, with good accuracy and free from complications or tilts. All VCFs were deployed in the infra renal portion of the IVC, via femoral vein access, with easy navigation and fluoroscopic observation.

The efficacy of clot capture, which was also observed with fluoroscopy, can be seen in Table 1.

**Table 1:** Capture efficacy and complication rates.

**Group**	**1**		**2**		**3**		**4**	
**Conditions**	**1A**	**1B**	**2A**	**2B**	**3A**	**3B**	**4A**	**4B**
Number of clots injected	0	0	2	2	4	4	8	8
Number of clots captured	0	0	2	2	4	3	4	4
Efficacy	-	-	100%	100%	100%	75%	50%	50%
Migration	No	No	No	No	No	No	No	No
Cava perforation	No	No	No	No	No	No	No	No
Inflammatory response	No	No	No	No	No	No	No	No
Explantation (days)	0	33	0	78	0	30	0	39

Group 2 capture efficacy was 100%. In group 3, the capture rate was 100% for one animal and 75% for the other. In group 4, capture efficacy was 50%. Clots that had not been captured by the VCF were located in pulmonary artery branches by fluoroscopy. Although clot capture was not 100% in some experiments, there were no clinical consequences for the animals during the procedure or during the observation period.

During follow-up, which varied from 30 to 78 days, B animals remained healthy and exhibited no complications related to implantation of the VCF or other adverse effects, such as bruising at the insertion site, infection or hemodynamic instability.

Cavographies were performed before IVC explantation. They showed patent IVCs in all animals and there was no evidence of damage to the blood vessel, device migration or VCF angle changes.

No adhesion of the VCF to the vessel was observed during histopathological analysis. The inner layer was composed of typical endothelial cells with presence of discrete hemorrhage and there was no inflammation or tissue calcification. The VCFs had not suffered deformation, deterioration, fracture or migration.

## DISCUSSION

The process of bringing a new VCF to the market requires a well-defined set of performance criteria and mathematical models to identify factors affecting device performance. Procedures should be designed to accurately simulate the results of a biological event.^9^ Therefore, a comprehensive evaluation of the device’s behavior in both in vitro scenarios and using animal experiments is necessary.

At a time in which there are concerns about animal experiments, it is essential that in vivo and in vitro studies be designed to obtain maximum benefit from limited resources. An accurate in vitro evaluation of VCF prototypes provides us with data showing that this product is, at least, comparable to designs already on the market, limiting use of animal resources and preventing exposure of patients to devices with low efficacy or important adverse effects.^9^

The device used in this study has previously been evaluated on an experimental bench where tests were performed in an artificial system. The filter selected exhibited the same capture efficacy as commercially available filters.^8^

Although the predictive value of in vitro models has significantly reduced the number of animal required for testing, certain features such as navigation, behavior upon release, thrombogenicity, endothelial reaction and migration can only be evaluated using in vivo tests. These tests allow us to judge safety and efficacy as well as preventing premature adoption of unsuitable products. Successful in vivo and in vitro tests are generally associated with successful clinical performance.^9^

Sheep are good animals to use in these experimental tests because the diameter of the ovine VCI is similar to the diameter in humans.^10^ Sheep were chosen for this study because of this similarity and because of the high correlations between in vivo studies with these animals and clinical trial results.

The BKone1 filter was designed to offer a high clot capture rate, to preserve IVC patency and to remain centered. Minimal changes in metal elasticity and the length or angle of rods or their components can significantly affect filter efficacy.

The experimental method allowed us to evaluate clot retention as well as other desired parameters. Using a metal mold to make standardized clots enabled better comparison between groups. The radiopaque markers inserted into these clots enabled fluoroscopic analysis of VCF capture efficacy.

In this study, all devices were positioned accurately and correctly oriented and the animals did not have any complications related to implementation of the VCFs. The same results have also been observed in other studies of commercially available VCFs.^1^^,^ 9

The results indicate that filter efficacy decreases with a higher embolic load, which has also been observed in other studies.^11^ However, these results can not be compared or extrapolated from one study to another because a standardized experimental protocol has not been defined. Despite the lower capture efficacy in groups with a higher embolic load, no sheep died during experiments or follow-up.

Use of a guide wire to deploy VCFs (over-the-wire method) ensures correct device positioning. In vitro studies have shown that changes in angle relative to the original position are associated with loss of clots and consequently lost capture capability.^12^ In this study, the devices remained centralized according to both control cavography and histopathological examination. Moreover, there was no deformation, fracture or migration, showing good material quality and good device design. Proctor et al.^9^ tested the Greenfield^TM^ vena cava filter in ovine models and compared it with other commercially available filters and reported similar results for these variables.

As was observed with the Greenfield^TM^ VCF and modified Greenfield filter,^9^^,^ 10 histopathological analysis of the BKone1 filter revealed good biocompatibility and incorporation of the VCF with limited vessel reaction, which is important for future development of a temporary device. Another important factor is IVC patency and this was observed in all animals, confirming low device material thrombogenicity.

## CONCLUSION

Overall, the experimental method used in this study proved adequate for assessing clot retention as well as other desired parameters. The BKone1 VCF demonstrated ease of deployment, resistance to migration and durability in an ovine model. Histopathological analysis showed good biocompatibility and incorporation. There was a reduction in efficacy with larger numbers of clots, but no clinical repercussions were observed.
